# Safety and organ health with 8 weeks use of commercially available bio-active peptide supplement: A prospective, double-blind, placebo controlled randomized trial

**DOI:** 10.1186/1550-2783-12-S1-P46

**Published:** 2015-09-21

**Authors:** Patrick L Jacobs

**Affiliations:** 1 Superior Performance Research, LLC; Miami, FL 33186, USA

## Background

It has been established that bio-active peptides may provide improved health, muscular performance and immune function. Enhancements in athletic performance and gains in lean muscle mass have also been reported with training. While the safety of bio-active peptides has been previously reported, it was the purpose of this investigation to examine the specific effects of a commercial bio-peptide product, Bio-Gro™, on blood markers of health and organ function and resting hemodynamic measures in men engaged in intense resistance training as indications of safety.

## Methods

Twenty recreationally resistance trained men voluntarily participated in this prospective, randomized, double-blind, placebo-controlled research investigation. Study participants were randomly assigned to receive two servings of either Bio-Gro™ bio-active peptides or placebo daily for an eight week study period. All study participants completed four intense weight training sessions weekly for the first four weeks and five intense weight training sessions performed per week for the final four weeks. Before and after the eight week program, assessment sessions were performed including standard complete blood counts, comprehensive metabolic panels, and resting hemodynamics. Measures were examined using two-way ANOVAs for repeated measures. Statistical significance was accepted at the p < 0.05 level.

## Results

Analyses of the blood chemistry count measures indicated significant main effects of group for RBC and significant main effects of time for values of MCV and MCHC (p values < 0.05). There were no statistically significant interaction effects (group × time) for any blood chemistry count measures indicating no specific effects of supplementation on these variables (p values > 0.05).

Results of the analyses of comprehensive metabolic panel measurements revealed significant effects of group for creatinine, eGFR, and BUN/creatinine (p values < 0.05). There were numerous significant main effects of time including BUN, BUN/creatinine, sodium, chloride, CO2, calcium, albumin, and alka phosphate (p values < 0.05). Analyses also showed no statistically significant interaction effects (group × time) for any comprehensive metabolic panel measures which indicated that Bio-Gro™ had not specific effects on the measures of the metabolic panels (p values > 0.05).

The analyses of resting hemodynamic measurements revealed significant time effects for HR (p < 0.05) with no significant effects of group or significant group × time interactions for HR, SBP, or DBP (p values < 0.05) again indicating that the supplementation had no specific effects on these variables.

## Conclusion

The results of the present study indicate that 56 days ingestion of a commercial bio-active peptide supplement, Bio-Gro™, produced no significant effects on complete blood count measures, values from comprehensive metabolic panels, or on resting hemodynamic measures in men participating in intense resistance training. These findings indicate that short-term supplementation of this bio-active peptide product is safe in apparently healthy, recreationally trained men when ingested at recommended dosages.

